# The Effect of Laparoscopic Sleeve Gastrectomy on Symptoms of Gastroesophageal Reflux Disease

**DOI:** 10.7759/cureus.31548

**Published:** 2022-11-15

**Authors:** Dalia Himika, Viktoriya Katsnelson, Isaac Alsallamin, Ameed Bawwab, Deema Chakhachiro

**Affiliations:** 1 Internal Medicine, St. Vincent Charity Medical Center, Cleveland, USA

**Keywords:** non-cardiac chest pain, bariatric surgery, morbid obesity, laparoscopic sleeve gastrectomy, gastroesophageal reflux

## Abstract

Background

Laparoscopic sleeve gastrectomy (LSG) is a common bariatric procedure for weight loss. LSG is becoming prevalent worldwide because it is a relatively simple procedure with high efficacy. Reduced intraabdominal pressure may improve gastroesophageal reflux disease (GERD) symptoms and reduce the GERD medication needed following LSG. However, the main long-term drawback of LSG is the development of de novo GERD. Therefore, we conducted this study to determine the relationship between GERD symptoms and LSG.

Methods

We conducted a retrospective chart review involving 390 patients who underwent LSG. Study participants were evaluated for GERD symptoms six months before and three, six, and nine months after the procedure, and proton-pump inhibitors (PPIs) were used to control the symptoms. Participants were distributed into two groups: one group for patients with GERD symptoms (36.1%) and one group for asymptomatic patients (62.8%). We collected demographic data and assessed PPI use in both groups after three, six, and nine months postoperatively. Data were collected using Microsoft Excel (Microsoft Corporation, Redmond, WA) and analyzed using IBM SPSS Statistics for Windows, Version 20.0 (Armonk, NY: IBM Corp.). We compared data using the student's t-test for independent groups. The quantitative data were summarized using mean and standard deviation (SD), and p < 0.05 was considered statistically significant.

Results

Of the 390 participants who underwent LSG, 83.8% were women (n=327) and 16.2% were men (n=63), with a median age of 42 ± 11.9 years. PPI use was statistically significantly greater after LSG (34.1%) than before LSG (24.6%, p=0.019). The difference in PPI use between symptomatic and asymptomatic groups was not statistically significant three months after LSG.

Conclusions

Our study focuses on using PPI after LSG due to GERD symptoms. We found GERD symptoms improved three months following LSG, but de novo GERD symptoms occurred nine months after the surgery. Health providers need to discuss with their patients the potential outcomes of the surgery and manage patient expectations. Physicians should work with their patients to assess whether the benefits of bariatric surgery in controlling overweight-associated conditions, such as blood pressure, diabetes, sleep apnea, and weight loss, outweigh the risk of GERD symptoms and PPI use.

## Introduction

Obesity and its related metabolic disorders are increasing in the US and worldwide [1]. Obesity increases the risk of associated diseases and worsening pre-existing diseases and increases the burden on the health care system and budgets. Obesity is a major risk factor for coronary artery disease, hypertension, diabetes, sleep apnea, hypopnea syndrome, gastroesophageal reflux disease (GERD), joint and bone disease, and arthritis [2]. Up to 50% of morbidly obese patients may suffer GERD [3].

Bariatric surgery has been proven to produce sustainable effects in morbidly obese patients with weight reduction and remission of comorbidities [4,5]. Laparoscopic sleeve gastrectomy (LSG) is one of the most common bariatric surgical procedures. Although it is a relatively recent introduction and lacks long-term results, it is a relatively simple technique with high efficacy. LSG reduces intraabdominal pressure due to weight loss, reduces acid production secondary to the acid-producing gastric fundus resection, accelerates gastric emptying, and reduces gastric volume. All these effects contribute to the reduction in gastric refluxate that putatively causes GERD symptoms [6,7]. However, a long-term adverse effect of LSG is the development of de novo GERD in some patients [8]. Therefore, this study aims to assess the relationship between de novo GERD and bariatric surgery by evaluating proton-pump inhibitor (PPI) use for GERD symptoms before LSG, GERD incidence after LSG, PPI use for GERD after LSG, the incidence of postoperative esophageal gastroscopy (EGD), and de novo GERD symptoms after LSG. EGD is a screening option for severe, intractable GERD symptoms to rule out an alternative diagnosis. We noted whether our study participants received EGD due to GERD symptoms post-surgery as an indicator of symptom severity.

## Materials and methods

We conducted a retrospective review of the medical records of 390 patients who underwent LSG at the Center for Bariatric Surgery at St. Vincent Charity Medical Center (SVCMC) in 2017. The study included patients referred for morbid obesity (body mass index (BMI) ≥ 40 kg/m^2^ or BMI ≥ 35 kg/m^2^ with obesity-related complications such as diabetes, hypertension, sleep apnea, or musculoskeletal problems) and scheduled for weight loss surgery. This research was approved by the Institutional Review Board for research and ethics at SVCMC (Approval No. 537).

The study excluded patients younger than age 18 and pregnant patients. Participants were distributed into two groups prior to LSG: one group for patients with GERD symptoms (36.1%) and one group for asymptomatic patients (62.8%). The study was conducted at a 95% confidence interval, and a t-test was used to determine if there was a significant between the two groups.

We reviewed patient charts for documented GERD symptoms six months before and three, six, and nine months after LSG. We noted GERD symptoms such as bloating, pain, satiety, and food tolerance/quality of alimentation. We also measured participant BMI and EGD before and after the LSG procedure, regardless of the indication for endoscopy.

We collected demographic data and assessed PPI use postoperatively after three, six, and nine months. Data were collected using Microsoft Excel (Microsoft Corporation, Redmond, WA) and analyzed using IBM SPSS Statistics for Windows, Version 20.0 (Armonk, NY: IBM Corp.). We compared data using the student's t-test for independent groups. The quantitative data were summarized using mean and standard deviation (SD), and p < 0.05 was considered statistically significant.

## Results

After applying the exclusion criteria, the study included 327 women (83.8%) and 63 men (16.2%). The median age was 42 ± 11.9 years. Before the LSG procedure, 118 patients had GERD symptoms (36.1%), and 209 were asymptomatic (62.8%) (Table 1).

**Table 1:** Participant sex data (N=390) Abbreviation: GERD, gastroesophageal reflux disease

Sex	Total, n (%)	No GERD Symptoms Before Surgery, n (%)	GERD Symptoms Before Surgery, n (%)	P-Value
Male	63 (16.2%)	38 (15.3%)	25 (17.7%)	0.57
Female	327 (83.8%)	211 (84.7%)	116 (83.3%)	

For patients without preoperative GERD, we found no significant difference between preoperative and postoperative PPI use (p=0.92). There was no significant difference between preoperative and postoperative PPI use when considering all patients (p=0.56; Table 2).

**Table 2:** GERD symptoms before surgery and the use of PPI Abbreviations: GERD, gastroesophageal reflux disease; PPI, proton-pump inhibitor

Preoperative GERD	No PPI Before Surgery, n (%)	PPI Before Surgery, n (%)	PPI After Surgery, n (%)	P-Value
No	10 (10.2%)	0 (0.0%)	232 (59.4%)	0.92
Yes	6 (1.53%)	96 (24.6%)	133 (34.1%)	0.019

Also, there were no significant differences in preoperative PPI use and three-month PPI, regardless of GERD status (Table 3).

**Table 3:** PPI three months after surgery Abbreviations: GERD, gastroesophageal reflux disease; PPI, proton-pump inhibitor

	No PPI Three Months After Surgery, n (%)	PPI Three Months After Surgery, n (%)	P-Value
No PPI Before Surgery	6 (0.01%)	271 (69.4%)	0.57
PPI Before Surgery	3 (0.76%)	92 (23.5%)	
No GERD Before Surgery (Asymptomatic)	3 (0.76%)	234 (60%)	0.97
GERD Before Surgery (Symptomatic)	3 (0.76%)	131 (33%)	

PPI use significantly declined between preoperative GERD and six-month postoperative GERD patients (p=0.049). For patients without preoperative GERD symptoms, the use of PPI did not significantly change following LSG. Among patients with preoperative GERD, 41.8% did not need PPI six months after LSG while 58.2% required PPI at six months. Two patients use antihistamine antacids due to PPI allergies (Table 4).

**Table 4:** PPI six months after surgery Abbreviations: GERD, gastroesophageal reflux disease; PPI, proton-pump inhibitor

	No PPI Six Months After Surgery, n (%)	PPI Six Months After Surgery, n (%)	P-Value
No GERD Before Surgery (Asymptomatic)	93 (23.8%)	107 (27.4%)	0.049
GERD Before Surgery (Symptomatic)	46 (11.7%)	64 (16.4%)	

Six months postoperatively, 16% of symptomatic GERD patients used PPI, and 27.4% of asymptomatic patients used PPI (p=0.49).

We noted a statistically significant decline in PPI use nine months postoperatively compared to preoperative PPI use in GERD patients (p=0.012). However, in patients without preoperative GERD, there was no significant difference in PPI use postoperatively (Table 5).

**Table 5:** PPI nine months after surgery Abbreviations: GERD, gastroesophageal reflux disease; PPI, proton-pump inhibitor

	No PPI Nine Months After Surgery, n (%)	PPI Nine Months After Surgery, n (%)	P-Value
No GERD Before Surgery (Asymptomatic)	127 (32.5%)	43 (11.0%)	0.012
GERD Before Surgery (Symptomatic)	55 (14.1%)	47 (12.05%)	

Comparing six-month and nine-month postoperative PPI use, we see that 7.5% of patients developed de novo GERD following LSG. Approximately 25% of patients required PPI at nine months postoperatively. Fifty-five patients received preoperative EGD, and 17 received postoperative EGD; the difference was not statistically significant (p > 0.99). Two patients had EGD both preoperatively and postoperatively.

One hundred ten patients (33.7%) had a BMI of > 50 kg/m^2^ (i.e., super morbidly obese) and GERD symptoms, and were on PPI therapy before the surgery. Two hundred sixteen patients (66.3%) had BMI < 50 kg/m^2^. Seven patients (77.8%) with a BMI < 50 kg/m^2^ and preoperative GERD had postoperative GERD at the three-month follow-up. Only two patients (22.2%) with BMI > 50 kg/m^2^ and preoperative GERD had postoperative GERD at the three-month follow-up.

One hundred fifty-five patients (90.6%) with a BMI < 50 kg/m^2^ and preoperative GERD had postoperative GERD at six months. Sixteen patients (9.4%) with a BMI > 50 kg/m^2^ and preoperative GERD had postoperative GERD at six months. By the nine-month follow-up, 180 patients (97.3%) with a BMI < 50 kg/m^2^ and preoperative GERD had postoperative GERD. Only five patients (2.7%) with a BMI > 50 kg/m^2^ and preoperative GERD had postoperative GERD. However, only seven of 117 patients with a BMI > 50 kg/m^2^ presented for the nine-month evaluation. No significant differences were found for patients with a BMI > 50 kg/m^2^ and at the nine-month follow-up for GERD symptoms compared to those with prior surgery, although we noted marginal significance (p=0.067) at six months (Table 6).

**Table 6:** Comparison of PPI use for patients according to BMI after LSG three, six, and nine months Abbreviations: BMI, body mass index; PPI, proton-pump inhibitor

PPI Use			BMI ≤ 50 kg/m^2^	BMI > 50 kg/m^2^	Total
Three-Month Postoperative	No	Count	7	2	9
		% PPI Use in Three Months	77.80%	22.20%	100.00%
	Yes	Count	323	44	367
		% PPI Use in Three Months	88.00%	12.00%	100.00%
	Total	Count	330	46	376
		% PPI Use in Three Months	87.80%	12.20%	100.00%
Six-Month Postoperative	No	Count	134	5	139
		% PPI Use in Six Months	96.40%	3.60%	100.00%
	Yes	Count	155	16	171
		% PPI Use in Six Months	90.60%	9.40%	100.00%
	Total	Count	289	21	310
		% PPI Use in Six Months	93.20%	6.80%	100.00%
Nine-Month Postoperative	No	Count	180	5	185
		% PPI Use in Nine Months	97.30%	2.70%	100.00%
	Yes	Count	88	2	90
		% PPI Use in Nine Months	97.80%	2.20%	100.00%
	Total	Count	268	7	275
		% PPI Use in Nine Months	97.50%	2.50%	100.00%

Fourteen patients (3.59%) were lost to follow-up at three months, 80 patients (20.51%) were lost to the six-month follow-up, and 115 patients (29.49%) were lost to the nine-month follow-up. A significantly higher no-show rate was noted for patients with BMI > 50 kg/m^2^ than those with a lower BMI. 

## Discussion

In our retrospective chart review, we assessed the effects of LSG on GERD symptoms in obese patients. GERD symptoms improved in the first three months after surgery if the patient had symptoms before surgery. However, after six to nine months, most patients developed new-onset GERD, regardless of their symptoms before the study, and some had severe symptoms that required the daily use of PPI.

Obesity increases the risk of GERD due to elevated intrabdominal pressure, loosening of the lower esophageal sphincter (LES), and histological changes of the gastroesophageal junction due to fat deposition. A vicious cycle of acid reflux to the esophagus and weak sphincters, plus weak abdominal muscle due to fat deposition, results in worsening GERD symptoms [9,10].

After reviewing the literature, the theories behind the pathophysiology of new-onset GERD after LGS are multifactorial. Due to the procedure, the stomach anatomy, the smaller size of the stomach, and the funnel shape favor acid reflux and GERD symptoms. Because the shape was manipulated, the decrease in LES pressure increases the risk of acid reflux and GERD symptoms [11].

Several studies report a significant increase in the incidence of GERD after LSG and worsening of underlying symptomatic GERD with more complications and esophagitis or Barrett's esophagus (Table 7) [12-18].

**Table 7:** Summary literature review Abbreviations: GERD, gastroesophageal reflux disease; LRYGB, laparoscopic Roux-en-Y gastric bypass; LSG, laparoscopic sleeve gastrectomy; ReSG, ReSleeve Gastrectomy; OAGB/MGB, one anastomosis gastric bypass/mini-gastric bypass

Author, Year	Study Design	Results	Recommendations
Studies that showed a significant increase in risk			
Felsenreich et al., 2018 [12]	Multi-center study, 10-year update	57% of participants developed reflux symptoms	Gastroscopy after five years to detect GERD and conversion to Roux-en-Y to cure the symptoms
Elkassem et al., 2021 [13]	Prospective, nonrandomized trial of 13 patients, three to four years of age	New-onset GERD developed in 30.9% of patients	Most patients had stable preoperative GERD and required close follow-up and screening after surgery for possible symptomatic GERD
Yeung et al., 2020 [14]	Meta-analysis of 46 studies with 10,718 patients	Postoperative risk of developing GERD increased by 19%, new-onset GERD developed in 23% of cases	The benefits of surgery overweight the risk of long-term complications as GERD and esophagitis still warranted good surveillance
Raj et al., 2019 [15]	Prospective, nonrandomized, open-label clinical trial of 46 patients followed for six months	Incidence of GERD was 66.6% after LGS	Avoid LGS if patient has symptoms of GERD before the surgery, use LRYGB
Noel et al., 2020 [16]	Prospective clinical trials of all patients with failure after LSG who underwent ReSG with five-year outcomes	GERD in two patients (3.8%)	Further studies needed to compare LSG with Roux-en-Y
Braghetto et al., 2021 [17]	Prospective study of 39 patients, without a history of GERD symptoms before surgery	Symptomatic GERD developed in 84.6% after LSG in 8 to 71 months.	Support conversion of LSG to LRYGB if symptomatic GERD
King et al., 2021 [18]	Systematic review of the literature	High incidence of GERD following LSG	Surgeons to be aware of GERD symptoms before bariatric surgery to choose the best surgical approach and be familiar with the GERD development post-surgery
Studies that did not show a significant increase in risk			
Del Genio et al., 2014 [19]	Prospective, nonrandomized, 25 consecutive patients, 13 months follow-up	No de novo GERD after 13 months	Improve the LSG procedure techniques and follow the proper steps to avoid complications
Musella et al., 2021 [20]	Prospective randomized open-label, controlled trial of 58 patients with 12-month follow-up	No significant difference in GERD incidence after LSG and OAGB/MGB, rate of esophagitis is higher after LSG than OAGB/MGB	If GERD symptoms, avoid LSG and use OAGB/MGB
Studies that showed GERD symptoms improved after LSG			
Elzouki et al., 2020 [21]	A systematic literature review and metanalysis	GERD symptoms may improve but Roux-en-Y yields better outcomes	Recommend Roux-en-Y procedure for bariatric patients with GERD symptoms, more favorable to prevent GERD symptoms

However, a few studies showed no significant increase in the incidence of GERD after LSG or a minimal increase with no significant symptoms but showed significant esophageal complications related to acid reflux or delayed gastric emptying after LSG [19,20]. Surprisingly, one study showed that symptoms of GERD may improve one year after LSG [21]. We found an increased risk of GERD and de novo GERD after LSG, and three-, six-, and nine-month follow-up interpretations of symptoms regarding BMI before and after surgery suggest that reduction of BMI was not the only factor associated with GERD symptoms.

Our study had some important limitations. This was a retrospective chart review with a small sample size, and many patients were lost to follow-up. Our study was also subject to confounding factors for GERD symptoms, over-the-counter PPIs or antacids, and recall bias. Also, given that several surgeons operated on these patients, it is reasonable to assume a certain amount of variation in skills with significant operator-dependent factors and some variation in LSG surgical techniques, and this applies to our study; that being mentioned, general rules and general techniques are the same for all patients in our study who underwent the same LSG procedure to minimize this error, and we have only two surgeons involved in our study, which makes this part very limited.

## Conclusions

GERD symptoms improved following LSG, but de novo GERD symptoms occurred nine months postoperatively. Our study showed improvement in GERD symptoms three months after the surgery, but they started to appear again after three months. Also of significant note is that patients who never had GERD symptoms start to have new-onset GERD after nine months.

Early recognition of symptoms can help patients avoid anxiety or frustration after surgery and invasive diagnostic procedures such as EGD. The importance of bariatric surgery is to control associated diseases, overweight, and the development of GERD, necessitating an open discussion with the patient before the procedure. If possible, an alternative procedure, such as Roux-en-Y with a lower risk for GERD to minimize this side effect, is a good option for high-risk patients and should be discussed appropriately with patients.
